# A comparative analysis of EGFR mutation status in association with the efficacy of TKI in combination with WBRT/SRS/surgery plus chemotherapy in brain metastasis from non-small cell lung cancer

**DOI:** 10.1007/s11060-014-1570-7

**Published:** 2014-08-07

**Authors:** Ling Cai, Jian-fei Zhu, Xue-wen Zhang, Su-xia Lin, Xiao-dong Su, Peng Lin, Kai Chen, Lan-jun Zhang

**Affiliations:** 1 Department of Radiation Oncology, Sun Yat-Sen University Cancer Center, Guangzhou, 510060 China; 2 Department of Thoracic Surgery, Sun Yat-Sen University Cancer Center, Guangzhou, 510060 China; 3 Department of Pathology, Sun Yat-Sen University Cancer Center, Guangzhou, 510060 China

**Keywords:** EGFR mutation, TKI, Brain metastasis, Non-small cell lung cancer

## Abstract

We proposed to identify the efficacy of an epidermal growth factor receptor (EGFR) tyrosine kinase inhibitor (TKI) using whole brain radiotherapy (WBRT)/stereotactic radiosurgery (SRS)/surgery in brain metastases from patients with non-small cell lung cancer (NSCLC) and clarify the association between treatment outcome and EGFR gene mutation status. A total of 282 patients with NSCLC brain metastases who underwent WBRT/SRS/surgery alone or in combination with TKI were enrolled in our study from 2003–2013. Amplification mutation refractory system technology was used to determine the EGFR mutation status in 109 tissue samples. EGFR mutation detection was performed in 109 patients with tumor tissues. The EGFR positive rate was 50 % (55/109), including 26 exon 19 deletions and 24 L858R mutations. The median follow-up time was 28 months. The median overall survival, median progression-free survival of intracranial disease, and median progression-free survival of extracranial disease was significantly longer for patients with TKI treatment (31.9 vs 17.0 months, *P* < 0.0001; 19.8 vs 12.0 months, *P* < 0.0001; and 19.6 vs 12.3 months, *P* < 0.0001; respectively). In subgroup analysis within the TKI group, patients harboring EGFR mutations had better extracranial disease control (20.4 vs 14.1 months, *P* = 0.032). Administration of TKI agents with conventional therapy compared with conventional therapy alone might be beneficial for overall survival, progression-free survival of intracranial disease and progression-free survival of extracranial disease in patients with brain metastases from NSCLC independent of EGFR mutations.

## Introduction

Non-small cell lung cancer (NSCLC) accounts for approximately 80 % of all lung cancers, in which brain metastases (BMs) occur in 20–40 % of all NSCLC cases and represent a major pattern of treatment failure and cause of mortality [1, 2]. Whole brain radiotherapy (WBRT) has been considered as a standard therapy for patients with BMs and leads to an overall survival (OS) ranging from 3 to 6 months [3, 4]. Although the standard management has been optimized over time with the development of stereotactic radiosurgery (SRS) combined with systemic chemotherapy and more accurate patient selection for appropriate treatment options now depends on a better definition of prognostic factors, outcomes of BM from NSCLC remain poor, with a short median survival time of 7–8 months [5].

Tyrosine kinase inhibitors (TKIs) of the epidermal growth factor receptor (EGFR) have been successfully employed in NSCLC based on the identification of EGFR gene mutations. Advances in understanding the molecular pathways that mediate brain colonization have led to a new interest and alternative to traditional therapy in clinical investigations in BMs from NSCLC [1, 6–9]. EGFR mutation status has been reported to be associated not only with improved survival for patients with BMs [10], but also with the response rate of WBRT [9]. More recently, TKIs have demonstrated a distinct therapeutic potential against BMs from NSCLC and have improved the median OS to 9–13.5 months [11–14]. Furthermore, a few studies reported an improved median OS of 13–23.4 months for BMs patients using TKIs concomitant or pretreated with WBRT [11, 15, 16].

Thus far, several studies have demonstrated the efficacy of using TKIs in patients with BMs from NSCLC. However, few studies have been published that discuss the relationship between EGFR gene mutation status and response rate for TKI administration simultaneously with WBRT/SRS/surgery in patients with BMs. In our study, we retrospectively reviewed 282 patients with BMs from NSCLC who received WBRT/SRS/surgery with or without TKI and detected the gene mutation status of tumor tissues from 109 patients. We proposed to investigate the efficacy of TKI in combination with traditional therapy and explore the relationship between EGFR gene mutation status and treatment efficacy in patients with BMs from NSCLC.

## Patients and materials

### Eligible patients

We retrospectively retrieved the data of 530 patients with brain metastasis from NSCLC treated in Sun Yat-sen University Cancer Center from 2003 to 2013. This study was approved for the use of tumor samples and patients’ clinical history by the Institutional Review Board. The main eligibility criteria were pathologically confirmed NSCLC and medical image measurable brain metastases. Consequently, a total of 282 patients treated with conventional therapy alone or in combination with TKI were included in this study. Conventional therapy included WBRT, SRS, or surgery (S), or a combination of these. All medical records were reviewed for age, gender, symptoms, physical examination, laboratory examination, imaging, pathological diagnosis, stage, biomarker detection, chest computed tomography, magnetic resonance imaging of the brain, modality of treatment, site and number of BMs, time to disease progression, time to death, and last follow-up date. Patients were treated according to our institute’s policy. All patients were grouped into TKI plus conventional therapy (TKI group) or conventional therapy alone (Non-TKI group).

### EGFR mutation detection

### Samples

Among all 282 patients, 109 (39 %) had adequate tumor tissue or lymph node biopsy samples for molecular analysis. The samples were formalin-fixed and paraffin-embedded (FFPE). All tumor specimens went through pathological evaluation to confirm the diagnosis of NSCLC and the percentage of tumor cells. As the analytical sensitivity of the ARMS method is approximately 1 %, at least 1 % of tumor cells were required for the following mutation detection.

### DNA extraction and quality check

The QIAamp DNA FFPE Tissue Kit (Qiagen, Hilden, Germany) was used for DNA extraction from tumor tissue samples following the instructions in the user manual. Extracted DNA samples were quantified by the real time quantitative PCR method using a commercial Taqman assay for the RNase P gene (Life Technologies, USA). The concentration of each DNA sample was normalized to 0.4 ng/µL whenever possible. When the DNA concentration was lower than 0.4 ng/µL, the original DNA stock solution was applied.

### EGFR mutation detection by the ARMS method

The EGFR Mutation Detection Kit (Amoy Diagnostics, Xiamen, China), which is based on the ARMS (amplification mutation refractory system) technology, was used to detect the 29 most common types of EGFR mutations and the T790 M mutation in lung cancer. All experiments were performed following the user manual. Briefly, 4.7 µL DNA was added to 35.3 µL PCR master mix, which contains PCR primers, fluorescent probes, PCR buffer, and Taq DNA polymerase. PCR thermal cycling was set as: 95 °C for 5 min, followed by 15 cycles of 95 °C for 25 s, 64 °C for 20 s, 72 °C for 20 s, and then 31 cycles of 93 °C for 25 s, 60 °C for 35 s, 72 °C for 20 s. Fluorescent signals were collected from the FAM and HEX channels. The results were analyzed according to the instructions from the user manual.

### Statistical methods

OS was calculated from the date of diagnosis to the date of last follow-up or death from any cause. Progression-free survival for intracranial disease (PFSI) was calculated from the date of diagnosis to the time to CNS-progression. Progression-free survival for extracranial disease (PFSE) was calculated from the date of diagnosis to the time to extracranial disease progression. Survival curves were constructed using the Kaplan–Meier method and differences were considered significant if the *p* value was less than 0.05 (two-tailed log-rank test). Multivariate analysis (Cox-model) was used to determine the independent prognostic factors. All prognostic factors identified in the univariate analyses with *P* values <0.20 were included in the multivariate analyses.

## Results

Patient and treatment characteristics are presented in Table 1.

**Table 1:** Clinical characteristic for patients

Variables	TKI	Non-TKI	*P* value
Age			0.1104
<65	91	141	
≥65	13	37	
Gender			0.3186
Male	62	118	
Female	42	60	
Smoking			0.5184
Yes	43	82	
No	61	96	
Histology			0.4329
AC	93	152	
Non-AC	11	26	
Intracranial symptom			1.21E−06
With	37	102	
Without	67	76	
BM number			0.027
<3	68	91	
≥3	36	87	
BM size			0.046
<3 cm	88	131	
≥3 cm	16	47	
T stage			0.1183
T0	18	18	
Others	86	160	
N stage			0.0004
N0	49	46	
Others	55	132	
EGFR mutation			0.2895
Positive	29	26	
Negative	21	33	
Unknown	56	119	
Distant metastases			0.9434
With	55	92	
Without	49	86	

The median age was 65 years (range 30–78 years). There were 180 males and 102 females included in the study. Adenocarcinoma was the dominant pathological subtype, occurring in 87 % of patients. Patients with intracranial symptoms were found more in the non-TKI group (36 %) than in the TKI group (13 %) (*P* = 1.21E−06). The non-TKI group had more patients with ≥3 BM numbers than the TKI group (*P* = 0.027).

Radiotherapy was performed on a majority of patients (70 %), in which WBRT accounted for 54 % and SRS for 16 %. Synchronous distant metastases were observed in 55 patients from the TKI group compared with 92 patients from the non-TKI group.

EGFR mutation analysis was performed in 109 (38 %) patients with tissue samples. In the patients where EGFR detection was performed, the rate of EGFR positivity was 26 % in the TKI group compared with 24 % in the non-TKI group. Among the 55 (55/109) patients with EGFR mutations, 26 were exon 19 deletions, 24 had L858R mutations, and 5 harbored mutations in exon 19 and L858R simultaneously. In addition, none of the tested samples were positive for the T790 M mutation. Patients having EGFR L858R point mutations had a longer but non-significant median overall survival (MOS), median progression-free survival for intracranial disease (MPFSI) and median progression-free survival for extracranial disease (MPFSE) compared to patients with exon 19 deletions (Table 2).

**Table 2:** EGFR mutation status in association with TKI treatment

EGFR mutation	MOS					MPFSI					MPFSE				
	TKI group		Non-TKI		*P* value	TKI		Non-TKI		*P* value	TKI		Non-TKI		*P* value
Positive	30.9		11.2		<0.0001	17.9		9.6		0.0004	16.3		9.0		<0.0001
Del-19	23.6	P	10.9	P		18.5	P	5.7	P		16.1	P	8.0	P	
L858R	32.8	0.55	13.7	0.49		16.3	0.89	10.9	0.75		17.0	0.67	9.3	0.41	
Negative	28.4		14.7		<0.0001	18.6		11.4		0.0032	12.7		10.4		0.0098

*MOS* median overall survival, *MPFSI* median progression-free survival of intracranial disease, *MPFSE* median progression-free survival of extracranial disease

**Fig. 1:**
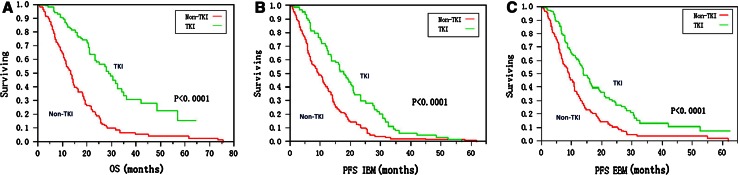
Survival curves in TKI and non-TKI groups, respectively. **a** Overall survival; **b** Progression-free survival of intracranial disease; **c** Progression-free survival of extracranial disease

With a median follow-up of 28 months (range, 22–34 months), 16 % (45) of patients were alive without evidence of disease progression, 17 % (48) were alive with disease, and 67 % (189) patients were dead due to disease progression. Overall, MOS, MPFSI, and MPFSE in the TKI group were 31.9 (95 % CI: 27.8–35.6), 19.8 (95 %CI: 16.8–22.8), and 19.6 (95 %CI: 16.4–22.8) months compared with 17.0 (95 % CI: 14.5–19.5) (*P* < 0.0001), 12.0 (95 %CI: 10.4–13.6) (*P* < 0.0001), and 12.3 (95 % CI: 10.4–14.2) (*P* < 0.0001) months in the non-TKI group, respectively (Fig. 1). A better outcome of MOS, MPFSI and MPFSE was observed in the TKI group compared to the non-TKI group independent from gene mutation status (Table 2).

Using univariate analyses, statistically significant factors favorably influencing MOS in the TKI group were as follows: patients with no extracranial disease, N0 stage, adverse drug reaction, and TKI taking time over 8 months. In the non-TKI group, less than 3 BMs number, less than 3 cm in maximum diameter of a BM lesion, no extracranial disease, T stage equal to or less than 1, and N0 were favorable factors influencing MOS (Table 3). For MPFSI, no significant factors were found in the TKI group, except for patients taking TKI over 8 months. However, patients with less than 3 cm in maximum diameter of BMs lesions or in N0 staging had better outcome in the non-TKI group (Table 3). For MPFSE, patients with EGFR mutations had a longer time to extracranial disease progression of 20.4 months compared with 14.1 months in EGFR negative patients (*P* = 0.032) in the TKI group. No difference could be found between EGFR positive and negative patients in the non-TKI group. The following parameters were in favor of MPFSE in the TKI group: BMs patients who underwent S/SRS, no extracranial disease, T stage ≤1, N0, adverse drug reaction, taking TKI more than 8 months, and age <54 (Table 3).

**Table 3:** Survival analysis (Log-rank test) according to clinical-pathological factors in NSCLC Patients with BM

Variable	TKI group (*N* = 104)			Non-TKI group (*N* = 178)		
	MOS	MPFSI	MPFSE	MOS	MPFSI	MPFSE
EGFR test (positive vs. negative)	NS	NS	S (*P* = 0.032)	NS	NS	NS
Age (<54years vs. ≥54years)	NS	NS	S (*P* = 0.014)	NS	NS	NS
Gender (female vs. male)	NS	NS	NS	NS	NS	NS
Smoking status (ever vs. never)	NS	NS	NS	NS	NS	NS
Histological type	NS	NS	NS	NS	NS	NS
Number of BM (cut off 3)	NS	NS	NS	S (*P* = 0.021)	NS	NS
Size of BM (cut off 3 cm)	NS	NS	NS	S (*P* = 0.012)	S (*P* = 0.025)	S (*P* = 0.032)
Treatment option (WBRT vs. S/SRS)	NS	NS	S (*P* = 0.011)	NS	NS	NS
Extracranial lesions (yes vs. no)	S (*P* = 0.0002)	NS	S (*P* = 0.013)	S (*P* = 0.003)	NS	NS
Intracranial symptoms (yes vs. no)	NS	NS	NS	NS	NS	NS
T staging (T ≤ 1 vs. T > 1)	NS	NS	S (*P* = 0.029)	S (*P* = 0.005)	NS	S (*P* = 0.007)
N staging (N0 vs N1 + 2 + 3)	S (*P* = 0.0003)	NS	S (*P* = 0.0005)	S (*P* = 0.0005)	S (*P* = 0.037)	NS
Adverse drug reaction (yes vs. no)	S (*P* = 0.0002)	NS	S (*P* = 0.0075)			
TKI taking time (cut off 8 ms)	S (*P* < 0.0001)	S (*P* = 0.0006)	S (*P* = 0.0013)			

*MOS* median overall survival, *MPFSI* median progression-free survival of intracranial disease, *MPFSE* median progression-free survival of extracranial disease, *NS* no significant, *S* significant

After multivariate analysis, the remaining common independent prognostic factor for OS, PFSI and PFSE was taking TKI over 8 months in the TKI group. In addition to this, no extracranial disease was also an independent factor for OS. Patients treated with S/SRS or in N0 stage favored PFSE in the TKI group. In the non-TKI group, never smoking, BMs number <3, BMs lesion size <3 cm, no extracranial disease and N0 were independent factors for OS (Table 4).

**Table 4:** Results of multivariate survival analyses for TKI group according to the cox regression model

Variables	TKI group						Non-TKI group					
	OS		PFSI		PFSE		OS		PFSI		PFSE	
	RR	*P* value	RR	*P* value	RR	*P* value	RR	*P* value	RR	*P* value	RR	*P* value
Extracranial lesions (yes/no)	0.62	0.0007	–	–	–	–	0.80	0.0037	–	–	–	–
TKI taking time (cut off 8 ms)	0.58	0.0001	0.41	0.0008	0.73	0.0106	–	–	–	–	–	–
Treatment option (WBRT vs. S/SRS)	–	–	–	–	0.69	0.0103	–	–	–	–	–	–
Smoking status (never)	–	–	–	–	–	–	0.82	0.0127	–	–	–	–
BM number (<3)	–	–	–	–	–	–	0.84	0.0247	–	–	–	–
BM size (<3 cm)	–	–	–	–	–	–	0.79	0.0120	–	–	–	–
T staging	–	–	–	–	–	–	–	–	–	–	0.81	0.0087
N staging	–	–	–	–	0.76	0.0281	0.65	0.0000	0.78	0.0011	0.79	0.0020

*OS* overall survival, *PFSI* progression-free survival of intracranial disease, *PFSE* progression-free survival of extracranial disease

## Discussion

The prognosis of patients with BMs from NSCLC remains poor even with optimized multi-modality treatments with WBRT plus SRS, surgery or chemotherapy. WBRT has been considered as a standard treatment option in patients with BMs from NSCLC [3, 4], but it causes neurotoxicity which leads to leukodystrophy. SRS or surgery could be an alternative option, but only for a small subset of patients with solid or oligo-lesions. However, either intracranial or extracranial disease advances rapidly even when BMs are well controlled with conventional therapy and become main patterns of treatment failure in this setting. On the other hand, it has been demonstrated previously that systemic chemotherapy is generally inactive against BMs [17]. This chemotherapeutic drug resistance has been shown to be caused by decreased penetration into the parenchyma because of the blood–brain barrier (BBB) [17]. However, this hypothesis has been challenged by both animal and clinical studies. These recent studies have revealed that the BBB is already leaky in BMs with tumors >0.25 mm in diameter, and BMs tumors are as sensitive to chemotherapeutic drugs as extracranial tumors in NSCLC [17]. Thus, the BBB no longer plays an important role in the multidrug resistance of BM, and the chemosensitivity in metastasis lesions seems to primarily dominate the responsiveness of chemotherapy [17].

Since TKI therapy has demonstrated a high response rate for EGFR mutation carriers in NSCLC, many previous studies intended to improve the survival of BMs patients from NSCLC using targeted agents in addition to conventional therapy [11, 12, 14, 17–20]. In early studies of molecularly targeted therapy in BMs from NSCLC, monotherapy of TKI agents (Gefitinib or Erlotinib) showed a distinct therapeutic potential against BMs [11, 21]. Cappuzzo et al. [21] were the first to report the possible activity of TKIs on BMs from NSCLC in a compassionate use program. Previously, an immunohistochemical and morphometric analysis in an experimental BMs model in mice identified various growth factors as positive regulators of angiogenesis [22, 23]. This discovery of molecules involved in angiogenesis promised new targeted agents in anticancer therapy for patients with BMs from NSCLC.

Then, a prospective study [11] of Gefitinib on BMs from NSCLC reported that the response rate (RR) of TKI use in BMs patients was 10 %, with a median duration of response of 13.5 months and MOS of 5 months. Another review of 15 BMs from NSCLC reported that the RR of TKI use in BMs was 60 %, which was similar to the primary lung tumor, with a median duration of response of 8.7 months [17]. The increased RR is most likely attributed to previous radiation therapy.

In our study, a total of 109 cases (39 %) with primary tumor tissues or lymph node samples underwent EGFR mutation detection. Among patients with sample detection, 46 % (50) were in the TKI group and 54 % (59) were in the non-TKI group. The EGFR mutation rate was 50 %, which was similar to our published data [24]. We showed a significant improved outcome for patients in the TKI group with MOS, MPFSI and MPFSE of 31.9, 19.8 and 19.6 months, respectively, compared to 17.0, 12.0 and 12.3 months in the non-TKI group. TKI agents were administered as first line treatment to the patients followed by WBRT/SRS/S, or in combination with chemotherapy. In our subset analysis regarding the EGFR mutation status in association with the efficacy of TKI agents, we found that TKI treatment was beneficial for BMs patients in regards to MOS, MPFSI and PFSE independent from the EGFR mutation status. In a previous prospective randomized study undertaken in Asian patients, the Iressa Pan-Asia study (IPASS) [25] demonstrated the superiority of Gefitinib as a first line treatment compared to chemotherapy for EGFR positive patients with respect to PFS. Similarly, WJTOG 3405, NEJ 002, OPTIMAL and Hirsch FR’s studies [26–29] confirmed the improved outcome of PFS of up to 18.2 months for Gefinitib or Erlotinib treatment compared to standard chemotherapy in patients with EGFR mutations. Furthermore, the EURTAC [30] study addressed the same findings of TKI use as a first line treatment in advanced NSCLC harboring EGFR mutant tumors among a non-Asian population. Our analysis showed a similar outcome for TKIs administered as a first line treatment followed with conventional therapy in BMs patients. Also, the subgroup analysis within the TKI group showed a superiority of PFSE in patients with EGFR positive mutations. However, a non-significant but potentially better outcome of OS and PFSI was observed in EGFR positive patients. Our findings suggested that TKI administration had a superior effect as a first line treatment on OS and PFS for patients with BMs from NSCLC, but the EGFR mutation status made no difference for OS and PFSI. This might be due to the interfering efficacy of following treatment with WBRT/S/SRS or chemotherapy, which also contributes to the response rate of either the primary tumor or metastatic lesions, particularly those with pathologic heterogeneity. On the other hand, the prolonged survival of PFSE indicated that there was increased efficacy of TKI agents on extracranial disease control for patients with EGFR positive mutations. This implied that the initial advantage of TKI treatment to patients with EGFR mutation suppresses the interference of chemotherapy.

So far, published data have shown a range of 133 days to 23.4 months for MOS and 141 days to 10.6 months for MPFS in BMs patients with concomitant treatment of TKI and WBRT [15, 16, 31, 32]. Our data showed a much longer MOS, MPFSI and MPFSE than previous studies, most likely due to delivering the TKI as a first line therapy.

However, recently published data from RTOG 0320 [31] failed to demonstrate the advantage of Erlotinib concomitantly administered with WBRT plus SRS in NSCLC patients with 1–3 brain metastases. This result was most likely due to the relatively small sample size and ineffectiveness of Erlotinib as doublet chemotherapy for systemic disease control [31]. Our results were quite different from the RTOG 0320 trial. The possible explanations for the differences in survival might be the following: (1) Neurotoxicity increased with the concomitant delivery of TKI with WBRT plus SRS. (2) Chemotherapy was delivered in sequence to patients in a combination with TKI in our study. The sequential administration of TKI and chemotherapy might enhance the control of systemic disease by either drug due to the potential anticancer ability against tumor heterogenicity, which was reflected in prolonged OS and PFSE. Published data from the FAST-ACT II trial [33] also showed a significant improvement in PFS with sequential administration of erlotinib following gemcitabine/platinum chemotherapy.

In conclusion, administration of TKI agents with conventional therapy might have a beneficial effect on MOS, MPFSI and MPFSE for patients with BMs from NSCLC compared to conventional therapy alone. Patients harboring EGFR mutations not only had significant improvement in PFSE with TKI plus conventional treatment compared to EGFR negative patients, but also had a non-significantly better outcome of OS and PFSI. This treatment strategy warrants further investigation in a prospective study.

## References

[1] Soffietti R, Trevisan E, Ruda R (2012). Targeted therapy in brain metastasis. Curr Opin Oncol.

[2] Andrews DW, Scott CB, Sperduto PW, Flanders AE, Gaspar LE, Schell MC, Werner-Wasik M, Demas W, Ryu J, Bahary JP, Souhami L, Rotman M, Mehta MP, Curran WJ (2004). Whole brain radiation therapy with or without stereotactic radiosurgery boost for patients with one to three brain metastases: phase III results of the RTOG 9508 randomised trial. Lancet.

[3] Diener-West M, Dobbins TW, Phillips TL, Nelson DF (1989). Identification of an optimal subgroup for treatment evaluation of patients with brain metastases using RTOG study 7916. Int J Radiat Oncol Biol Phys.

[4] Borgelt B, Gelber R, Kramer S, Brady LW, Chang CH, Davis LW, Perez CA, Hendrickson FR (1980). The palliation of brain metastases: final results of the first two studies by the Radiation Therapy Oncology Group. Int J Radiat Oncol Biol Phys.

[5] Moscetti L, Nelli F, Felici A, Rinaldi M, De Santis S, D’Auria G, Mansueto G, Tonini G, Sperduti I, Pollera FC (2007). Up-front chemotherapy and radiation treatment in newly diagnosed nonsmall cell lung cancer with brain metastases: survey by outcome research network for evaluation of treatment results in oncology. Cancer.

[6] Eichler AF, Chung E, Kodack DP, Loeffler JS, Fukumura D, Jain RK (2011). The biology of brain metastases-translation to new therapies. Nat Rev Clin Oncol.

[7] Fidler IJ (2011). The role of the organ microenvironment in brain metastasis. Semin Cancer Biol.

[8] Preusser M, Capper D, Ilhan-Mutlu A, Berghoff AS, Birner P, Bartsch R, Marosi C, Zielinski C, Mehta MP, Winkler F, Wick W, von Deimling A (2012). Brain metastases: pathobiology and emerging targeted therapies. Acta Neuropathol.

[9] Gow CH, Chien CR, Chang YL, Chiu YH, Kuo SH, Shih JY, Chang YC, Yu CJ, Yang CH, Yang PC (2008). Radiotherapy in lung adenocarcinoma with brain metastases: effects of activating epidermal growth factor receptor mutations on clinical response. Clin Cancer Res.

[10] Eichler AF, Kahle KT, Wang DL, Joshi VA, Willers H, Engelman JA, Lynch TJ, Sequist LV (2010). EGFR mutation status and survival after diagnosis of brain metastasis in nonsmall cell lung cancer. Neuro Oncol.

[11] Ceresoli GL, Cappuzzo F, Gregorc V, Bartolini S, Crino L, Villa E (2004). Gefitinib in patients with brain metastases from non-small-cell lung cancer: a prospective trial. Ann Oncol.

[12] Hotta K, Kiura K, Ueoka H, Tabata M, Fujiwara K, Kozuki T, Okada T, Hisamoto A, Tanimoto M (2004). Effect of gefitinib (‘Iressa’, ZD1839) on brain metastases in patients with advanced non-small-cell lung cancer. Lung Cancer.

[13] Takahashi H, Ohrui T, Ebihara S, Yamada M, Sasaki H (2004). Effect of gefitinib (ZD1839) on metastatic brain tumour. Lung Cancer.

[14] Kim JE, Lee DH, Choi Y, Yoon DH, Kim SW, Suh C, Lee JS (2009). Epidermal growth factor receptor tyrosine kinase inhibitors as a first-line therapy for never-smokers with adenocarcinoma of the lung having asymptomatic synchronous brain metastasis. Lung Cancer.

[15] Zeng YD, Zhang L, Liao H, Liang Y, Xu F, Liu JL, Dinglin XX, Chen LK (2012). Gefitinib alone or with concomitant whole brain radiotherapy for patients with brain metastasis from non-small-cell lung cancer: a retrospective study. Asian Pac J Cancer Prev.

[16] Ma S, Xu Y, Deng Q, Yu X (2009). Treatment of brain metastasis from non-small cell lung cancer with whole brain radiotherapy and Gefitinib in a Chinese population. Lung Cancer.

[17] Namba Y, Kijima T, Yokota S, Niinaka M, Kawamura S, Iwasaki T, Takeda Y, Kimura H, Okada T, Yamaguchi T, Nakagawa M, Okumura Y, Maeda H, Ito M (2004). Gefitinib in patients with brain metastases from non-small-cell lung cancer: review of 15 clinical cases. Clin Lung Cancer.

[18] Fekrazad MH, Ravindranathan M, Jones DV (2007). Response of intracranial metastases to erlotinib therapy. J Clin Oncol.

[19] Popat S, Hughes S, Papadopoulos P, Wilkins A, Moore S, Priest K, Meehan L, Norton A, O’Brien M (2007). Recurrent responses to non-small cell lung cancer brain metastases with erlotinib. Lung Cancer.

[20] Porta R, Sanchez-Torres JM, Paz-Ares L, Massuti B, Reguart N, Mayo C, Lianes P, Queralt C, Guillem V, Salinas P, Catot S, Isla D, Pradas A, Gurpide A, de Castro J, Polo E, Puig T, Taron M, Colomer R, Rosell R (2011). Brain metastases from lung cancer responding to erlotinib: the importance of EGFR mutation. Eur Respir J.

[21] Cappuzzo F, Calandri C, Bartolini S, Crino L (2003). ZD 1839 in patients with brain metastases from non-small-cell lung cancer (NSCLC): report of four cases. Br J Cancer.

[22] Reijneveld JC, Voest EE, Taphoorn MJ (2000). Angiogenesis in malignant primary and metastatic brain tumors. J Neurol.

[23] Risau W (1997). Mechanisms of angiogenesis. Nature.

[24] Zhang LJ, Cai L, Li Z, Wang WP, Guo K, Shao JY, Wang JY, Yu H, Rong TH (2012). Relationship between epidermal growth factor receptor gene mutation and copy number in Chinese patients with non-small cell lung cancer. Chin J Cancer.

[25] Mok TS, Wu YL, Thongprasert S, Yang CH, Chu DT, Saijo N, Sunpaweravong P, Han B, Margono B, Ichinose Y, Nishiwaki Y, Ohe Y, Yang JJ, Chewaskulyong B, Jiang H, Duffield EL, Watkins CL, Armour AA, Fukuoka M (2009). Gefitinib or carboplatin-paclitaxel in pulmonary adenocarcinoma. N Engl J Med.

[26] Mitsudomi T, Morita S, Yatabe Y, Negoro S, Okamoto I, Tsurutani J, Seto T, Satouchi M, Tada H, Hirashima T, Asami K, Katakami N, Takada M, Yoshioka H, Shibata K, Kudoh S, Shimizu E, Saito H, Toyooka S, Nakagawa K, Fukuoka M (2010). Gefitinib versus cisplatin plus docetaxel in patients with non-small-cell lung cancer harbouring mutations of the epidermal growth factor receptor (WJTOG3405): an open label, randomised phase 3 trial. Lancet Oncol.

[27] Maemondo M, Inoue A, Kobayashi K, Sugawara S, Oizumi S, Isobe H, Gemma A, Harada M, Yoshizawa H, Kinoshita I, Fujita Y, Okinaga S, Hirano H, Yoshimori K, Harada T, Ogura T, Ando M, Miyazawa H, Tanaka T, Saijo Y, Hagiwara K, Morita S, Nukiwa T (2010). Gefitinib or chemotherapy for non-small-cell lung cancer with mutated EGFR. N Engl J Med.

[28] Zhou C, Wu YL, Chen G, Feng J, Liu XQ, Wang C, Zhang S, Wang J, Zhou S, Ren S, Lu S, Zhang L, Hu C, Luo Y, Chen L, Ye M, Huang J, Zhi X, Zhang Y, Xiu Q, Ma J, You C (2011). Erlotinib versus chemotherapy as first-line treatment for patients with advanced EGFR mutation-positive non-small-cell lung cancer (OPTIMAL, CTONG-0802): a multicentre, open-label, randomised, phase 3 study. Lancet Oncol.

[29] Hirsch FR, Kabbinavar F, Eisen T, Martins R, Schnell FM, Dziadziuszko R, Richardson K, Richardson F, Wacker B, Sternberg DW, Rusk J, Franklin WA, Varella-Garcia M, Bunn PA, Camidge DR (2011). A randomized, phase II, biomarker-selected study comparing erlotinib to erlotinib intercalated with chemotherapy in first-line therapy for advanced non-small-cell lung cancer. J Clin Oncol.

[30] Rosell R, Carcereny E, Gervais R, Vergnenegre A, Massuti B, Felip E, Palmero R, Garcia-Gomez R, Pallares C, Sanchez JM, Porta R, Cobo M, Garrido P, Longo F, Moran T, Insa A, De Marinis F, Corre R, Bover I, Illiano A, Dansin E, de Castro J, Milella M, Reguart N, Altavilla G, Jimenez U, Provencio M, Moreno MA, Terrasa J, Munoz-Langa J, Valdivia J, Isla D, Domine M, Molinier O, Mazieres J, Baize N, Garcia-Campelo R, Robinet G, Rodriguez-Abreu D, Lopez-Vivanco G, Gebbia V, Ferrera-Delgado L, Bombaron P, Bernabe R, Bearz A, Artal A, Cortesi E, Rolfo C, Sanchez-Ronco M, Drozdowskyj A, Queralt C, de Aguirre I, Ramirez JL, Sanchez JJ, Molina MA, Taron M, Paz-Ares L (2012). Erlotinib versus standard chemotherapy as first-line treatment for European patients with advanced EGFR mutation-positive non-small-cell lung cancer (EURTAC): a multicentre, open-label, randomised phase 3 trial. Lancet Oncol.

[31] Sperduto PW, Wang M, Robins HI, Schell MC, Werner-Wasik M, Komaki R, Souhami L, Buyyounouski MK, Khuntia D, Demas W, Shah SA, Nedzi LA, Perry G, Suh JH, Mehta MP (2013). A phase 3 trial of whole brain radiation therapy and stereotactic radiosurgery alone versus WBRT and SRS with temozolomide or erlotinib for non-small cell lung cancer and 1 to 3 brain metastases: radiation Therapy Oncology Group 0320. Int J Radiat Oncol Biol Phys.

[32] Lind JS, Lagerwaard FJ, Smit EF, Senan S (2009). Phase I study of concurrent whole brain radiotherapy and erlotinib for multiple brain metastases from non-small-cell lung cancer. Int J Radiat Oncol Biol Phys.

[33] Mok TS, Wu YL, Yu CJ, Zhou C, Chen YM, Zhang L, Ignacio J, Liao M, Srimuninnimit V, Boyer MJ, Chua-Tan M, Sriuranpong V, Sudoyo AW, Jin K, Johnston M, Chui W, Lee JS (2009). Randomized, placebo-controlled, phase II study of sequential erlotinib and chemotherapy as first-line treatment for advanced non-small-cell lung cancer. J Clin Oncol.

